# Building failure into successful team science: highlights of the second SINA*Innovations* event

**DOI:** 10.1242/dmm.015776

**Published:** 2014-03

**Authors:** Arielle Klepper, Matthew Pendleton

**Affiliations:** Icahn School of Medicine at Mount Sinai, 1428 Madison Avenue, New York, NY 10029, USA.

How does a scientist produce research results? The answer to the question has changed dramatically over time. Funding is a scarce commodity in the contemporary research world, and this represents a major challenge to harnessing the full potential of human ingenuity in the life sciences. Scientists are faced with government shutdowns, such as the US-based shutdown in late 2013 that had a major impact on researchers, disrupting grant application machinery and functionality of PubMed, a resource used by virtually every biomedical scientist in the world. The face of progress in biological sciences is clearly shifting; according to Thomson Reuters, the average number of authors on papers in the Science Citation Index has increased by 50% between 1990 and 2010. To address the increasing demand for funding, among other challenges, science research is increasingly being performed in teams, from multi-center clinical trials to multi-disciplinary teams working to model the diseases; teams are at the forefront of innovation.

Driven by this growing trend of team science, the Icahn School of Medicine at Mount Sinai sought to highlight “the power of Team Science, which is a key ingredient to accelerating progress and spurring creativity as we advance our culture of innovation and discovery” (ichan.mssm.edu/sinainnovations). The theme of team science formed the basis of the second annual SINA*Innovations* meeting, held in New York, NY at Icahn School of Medicine at Mount Sinai on 18 and 19 November 2013.

The aim of this event was to address the question: “how can strategies to improve teamwork promote innovation?”. The ‘team science’ model has long been in place in fields such as high-energy particle physics and astronomy, but is arguably more foreign to the realm of biological research. The program kicked off with Nirav Shah, who emphasized that a team need not be discrete. In his role as Commissioner of Health for New York, his success hinged on the fact that he had a team of people whose goal was to shape the health of all of New York, which meant that he had to engender the interest of a much larger team of citizens through an understanding of the sociology of large diverse groups.

Following that, Joe Torre spoke about his experience managing the New York Yankees. Joe Torre was the subject of a recent *Harvard Business Review* article: ‘Leadership that gets results’. He was described as an “affiliative leader”, with a skill of putting the team before himself. Joe Torre described how he instilled a mutual accountability into players who might otherwise be more concerned with being in the limelight. He placed a strong emphasis on how as the manager of a team, he was the buffer between upper management and his players, ensuring his team that he would always represent their best interests above all else. He described how his players came to understand that he would never portray them negatively in the media. This highlighted the idea that a team manager holds a great deal of power over the members, who must be reassured that this power would never be used underhandedly. Joe Torre also emphasized that learning how to fail, and how to rebound from failure, was key to the success of a team.

In the panel discussion that followed, the theme of allowing for failure in order to promote success was further emphasized. Jon Gertner, one of the panelists, authored *The Idea Factory* – the story of how the need to develop a national telephone infrastructure led to the formation of a fluid team, which produced some of the greatest inventions of the 20th century. He noted that often, “the single most important element to success at endeavors of innovation, team-based or otherwise, is time, autonomy, support and permission to fail to achieve a very specific goal”. This idea of having room to fail echoed throughout the rest of the talks.

**Figure:**
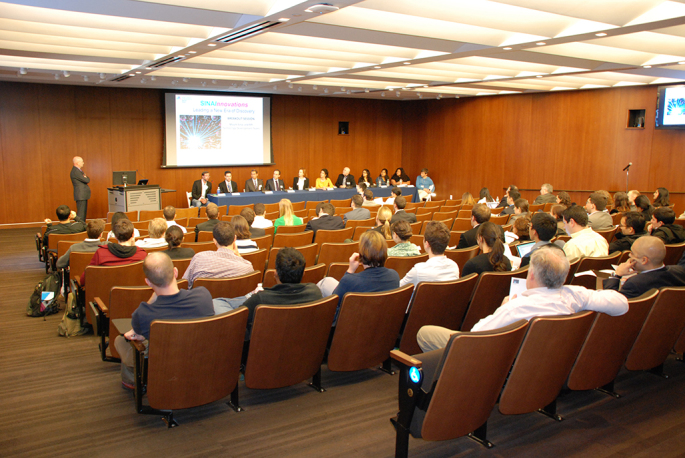


Moreover, there was talk of the constant struggle of reconciliation between this value of failure and the lack of room for failure built into the culture of science and in the funding structures in American science. This is a particularly “wicked problem” that was expounded by Noshir Contractor, Professor of Behavioral Sciences, Northwestern University, who described how failures in a team context often bring down the enthusiasm of other team members. He emphasized that a working solution often requires a large consensus to make grand drastic changes that conflict with existing interests. He also described his research into the assembly and maintenance of productive groups. He described his study of grant proposals submitted to the National Science Foundation, where he investigated the relationship between prior citation (an indicator of collaboration) and likelihood of being awarded the grant. He found that the grants most likely to be funded were from those teams that had lower levels of prior citation relationships. This finding suggests that teams composed of individuals with relatively low overlap in areas of expertise were more likely to succeed.

Dr Gary May, Dean of the College of Engineering at Georgia Institute of Technology, gave a keynote address. Dr May spoke of his institution’s marked success in generating licensed technologies. He reinforced the concept that their success was rooted in providing structure and funding for collaborations between diverse research specialties that might not otherwise overlap. Dr May also placed emphasis on the importance of creating diverse teams from the perspective of gender, race and culture.

The final keynote speaker, Dr Sara Diamond, President of Ontario College of Art & Design (OCAD) University, concluded the conference. Dr Diamond’s remarks underscored many of the concepts raised by previous speakers. She focused her remarks on evidence-based design, suggesting that, in order to drive innovation, teams at OCAD were focused on identifying gaps in product design that impacted underserved populations. In addressing these challenges, interdisciplinary teams were assembled, which was key to their success in addressing how to improve failed design strategies.

In a certain sense, the National Institutes of Health (NIH) as a meta-team of researchers in pursuit of a common goal of improving health outcomes is not very successful at promoting the brand of team science promoted at SINA*Innovations* in which failure is rewarded and diverse teams of scientists dominate. In addition to the obvious intolerance to failure shown at grant study sessions, it was pointed out that the pressures that publishers place on scientists are also to blame, a sentiment that has been echoed by Randy Scheckman since his Nobel recognition in December 2013, and recently discussed in a *Disease Models & Mechanisms* editorial (Matosin et al., 2014).

The 2013 SINA*Innovations* meeting made it clear that we need to evaluate and fix the shortcomings in how science as a team endeavor is organized. Although many speakers at SINA*Innovations* agreed that promoting diversity and making room for failure was key to the success of team science, and to promoting innovation, we are still left with the 100-billion-dollar question: where do we go from here? Does the key to fostering innovation lie with government funding sources such as NIH or the National Science Foundation (NSF)? Does it hinge upon a constellation of top-tier publishers and even tenure committees? Or is it central to start by incentivizing better cross-talk and partnership between academia and diverse industries?

Ultimately, a small group of scientists has a huge hand in deciding what grants get funded, what studies are published in peer-review journals and who gets a steady job in academia. The blame for failures of the team science on the largest scales inexorably belongs to scientists, even those who loudly object to those failures. However, although it’s easy to point out all the ways in which science falls short, this isn’t simply an exercise in criticism. We might fail repeatedly, but hopefully these failures become opportunities to improve, shedding light on how we can come together as a team to execute our Promethean goals… a discussion to be continued at next year’s SINA*Innovations*.

## Supplementary Material

Video Interviews
