# Supplementary material for: Building failure into successful team science: highlights of the second SINAInnovations event
Source: Dis Model Mech. 2014 Mar;7(3):311–2. doi: 10.1242/dmm.015776 (PMC3944490; doi:10.1242/dmm.015776)
Supplement: Video Interviews [file supp_7_3_311__index.html]

Video Interviews 

# Building failure into successful team science: highlights of the second SINA*Innovations* event

## DMM015776 Supplementary Material

Interview 1. Balancing Creativity & Profit in Innovation 1

Arielle Klepper, student at Mount Sinai School of Medicine, interviews Micheal Bierut, Partner at Pentagram, Rosalyn Taylor O'Neale, Principal Consultant at Cook Ross Inc., and Jon Gertner, author of 'The Idea Factory'.

Interview 2. Balancing Creativity & Profit in Innovation 2

Arielle Klepper, student at Mount Sinai School of Medicine, interviews Gordon Edelstein, Artistic Director at Long Wharf Theater.

Interview 3. Success in Team Science 1

Arielle Klepper and Matthew Pendleton, students at Mount Sinai School of Medicine, interview Alice Park, Staff Writer at TIME magazine.

Interview 4. Success in Team Science 2

Arielle Klepper, student at Mount Sinai School of Medicine, interviews Rear Admiral Scott P. Moore, Deputy Commander, Naval Special Warfare Command, US Navy.

Interview 5. Building the Infrastructure needed for Team Science

Matthew Pendleton, student at Mount Sinai School of Medicine, interviews Noshir Contractor, Northwestern University.

Interview 6. Art, Design & Medicine

Arielle Klepper and Matthew Pendleton, students at Mount Sinai School of Medicine, interview Sara Diamond, President, OCAD University.
